# Prevalence, Risk Factors, and Relationship between Reproductive Performance and the Presence of Antibodies against Coxiellosis in Dairy Farm Milk Tanks in the Northwest of Spain

**DOI:** 10.3390/ani14030367

**Published:** 2024-01-23

**Authors:** Uxía Yáñez, Jacobo Álvarez, Cristina Pisón, Antía Acción, Juan J. Becerra, Antonio Jiménez, Philippe Gisbert, Pedro G. Herradón, Ana I. Peña, Alberto Prieto, José M. Díaz-Cao, Luis A. Quintela

**Affiliations:** 1 Unit of Reproduction and Obstetrics, Department of Animal Pathology, Faculty of Veterinary Medicine, Campus Terra, Universidade de Santiago de Compostela, Avda. Carballo Calero s/n, 27002 Lugo, Spain; uxia.yanez.ramil@usc.es (U.Y.); jacobo.alvarez.torres@rai.usc.es (J.Á.); cristina.pison@rai.usc.es (C.P.); antia.accion.carro@rai.usc.es (A.A.); juanjose.becerra@usc.es (J.J.B.); garcia.herradon@usc.es (P.G.H.); ana.pena@usc.es (A.I.P.); 2 IBADER, Campus Terra, Universidade de Santiago de Compostela, Avda. Carballo Calero s/n, 27002 Lugo, Spain; alberto.prieto@usc.es (A.P.); josemanueldiaz.cao@usc.es (J.M.D.-C.); 3 CEVA SALUD ANIMAL S.A., Avda. Diagonal 609-615, 08028 Barcelona, Spain; antonio.jimenez@ceva.com; 4 CEVA SANTE ANIMALE, Avenue de la Ballastière 10, 33500 Libourne, France; philippe.gisbert@ceva.com; 5 INVESAGA Group, Department of Animal Pathology, Faculty of Veterinary Medicine, Campus Terra, Universidade de Santiago de Compostela, Avda. Carballo Calero s/n, 27002 Lugo, Spain

**Keywords:** *Coxiella burnetii*, Q fever, dairy cow, conception rate, ELISA, incidence, determinant factor

## Abstract

**Simple Summary:**

Q fever is an infectious disease caused by *Coxiella burnetii* that can affect both humans and animals. Given its consequences and the lack of epidemiological data published about its distribution and risk factors, we aimed to evaluate the prevalence of *Coxiella burnetii* at dairy farms in the northwest of Spain, identify which risk factors favor its occurrence, and the consequences on reproductive performance at the farm level. Bulk tank milk samples were collected from 262 farms and analyzed to identify antibodies against this bacterium. Additionally, data about potential risk factors and reproductive performance were obtained. A total of 60.1% of the farms tested positive for coxiellosis, and the main risk factors were the herd size, the purchase of livestock, and the geographical area. Additionally, conception rate and first-service conception rates were lower in positive farms, which also tended to have higher incidence of reproductive disorders after calving.

**Abstract:**

Q fever is a zoonotic disease that has been associated with reproductive problems in animals. As there is little epidemiological data regarding the distribution and risk factors of this disorder in cattle, the objective of this study was to evaluate the prevalence of *Coxiella burnetii* among dairy herds in the northwest of Spain, and to determine the on-farm risk factors associated with the disease and its effects on reproductive performance. Bulk tank milk (BTM) samples were collected from 262 commercial dairy herds from A Coruña, Lugo, and Pontevedra provinces. Data about location, mean age, and herd management features were obtained. A commercial indirect ELISA kit was used to determine the presence of antibodies against *C. burnetii* in BTM samples. The relationship between seropositivity to *C. burnetii* and the risk factors was checked using a Pearson’s χ^2^ test and a classification tree analysis. In addition, a one-way ANOVA test and the Mann–Whitney U test were used to check the impact of seropositivity to *C. burnetii* on reproductive performance. A total of 60.1% of the farms tested positive for coxiellosis, the herd size, the external purchase of livestock, and the geographical area were identified as the main risk factors. Conception rate and first-service conception rate were significantly lower (*p* < 0.05) in positive farms (37.1 and 32.9%) compared to negative farms (39.8 and 36.1%). Similarly, positive farms had significant higher incidence of endometritis (13.7% vs. 11.2%, *p* < 0.05). Consequently, a high seropositivity and slightly negative effects of coxiellosis on reproductive performance were observed, which intensifies the need for further research, including the identification an active infection in positive herds and the characterization of the genotype.

## 1. Introduction

Q fever is a zoonotic disease caused by an obligatorily intracellular bacterium, *Coxiella burnetii* [1], and nowadays it is considered endemic worldwide, with the exceptions of New Zealand and Antarctica [2,3]. The disease was firstly referred to a febrile illness observed in abattoir workers in Australia [4,5], and the term Q fever was also adapted in veterinary medicine, despite causing a different clinical course in animals. Although this terminology has been maintained, it has been suggested that coxiellosis may be more appropriate [5]. This bacterium presents a “smooth-rough” antigenic variation related to changes in the lipopolysaccharide (LPS) chain [6,7]. Thereby, two phases can be differentiated: cells in phase I, corresponding to soft antigenic variations, are highly infective and found in naturally infected humans, animals, and arthropods; and cells in phase II are harder and less infective variations that are obtained after serial passages in non-immune competent host systems [8]. The bacteria can be presented in three different variants: large cells or LCV (large-cell variants), small cells or SCV (small-cell variants), and small dense cells or SDC. The different forms are related to the biological cycle of the bacteria and their survival. *C. burnetii* is highly resistant to heat, drying, and many disinfectants, allowing this bacterium to survive for long periods in the environment [9].

It should be noted that numerous species of arthropods, birds, and mammals can be infected [3], although the primary reservoirs of *C. burnetii* are ticks, sheep, goats, and cattle. The placental tissue of infected animals may contain a large amount of *C. burnetii,* which can also be excreted in milk, urine, feces, semen, and amniotic fluids [3,10,11]. Humans are mainly infected by inhalation of contaminated dust [12]. Vertical and sexual transmission has also been reported [12,13], but is uncommon. Oral transmission of the bacteria via contaminated dairy products, although considered as negligible, has also been cited in the literature [14,15]. Additionally, the importance of cattle, goats, and sheep as sources of human infection may vary throughout the world. For example, data from England suggest that exposure to cattle, but not sheep, goats, cats, raw milk, or hay (all considered possible sources of Q fever) was associated with testing positive for *C. burnetii* immunoglobulin G (IgG) [16]. The authors of that study concluded that the risk of coxiellosis in livestock farms is related to contact with farm environment rather than any specific animal exposure [16]. In contrast, a literature review from the USA suggests that *C. burnetii* is enzootic in domestic ruminants and wild animals with widespread human exposure [17]. In eastern Asturias, in the north of Spain, sheep play the most relevant role in the transmission of *C. burnetii* to humans, but in the west of this autonomous community, cattle are the most important transmitter [18]. This fact results in the prevalence of the disease oscillating in this region between east and the west, being stable throughout the year in the western region and having a peak after the ovine lambing season in the east [18]. On the other hand, in Pakistan, small ruminants and camels have an important role in the transmission of this disease to humans [14].

In humans, Q fever infection is asymptomatic most of the time, but it can induce acute (flu-like illness, pneumonia, or hepatitis) or chronic (fatigue syndromes, endocarditis, or focalized infections) disease [11,12,19]. On the contrary, animals infected by *C. burnetii* rarely exhibit signs of disease, but the infection has been associated with an increase in the number of abortions, stillbirths, the birth of a weak calf, and infertility problems [20,21]. Apart from the control of *C. burnetii* infection in ruminants as a vital component of public health [9,10], given the important role of reproductive efficiency in dairy farm profitability, it is necessary to shed light on the negative effects of coxiellosis in cattle reproduction. 

Taking this information into account, one main problem associated with coxiellosis is that there is very little detailed epidemiological data regarding its distribution and risk factors in cattle from anywhere in the world. The seroprevalence rates reported in cattle populations vary greatly, ranging from 0.0% to 97.2% [10,22,23]. It is worth mentioning that Spain has a higher incidence of Q fever in comparison to other European countries, with the majority of diagnosed cases reported in northern Spain, where there is a greater concentration of livestock activities [20]. Moreover, the recent increase in interest in *C. burnetii* infection is concomitant with the fast and drastic improvement of diagnosis techniques, such as ELISA and PCR [1]. These are the most common methods used to identify *C. burnetii* presence in raw milk. In contrast to quantitative real-time PCR, ELISA is a good and reasonably priced indicator of the seroprevalence of coxiellosis; although, it cannot detect shedders, and it appears to be more sensitive for detecting antibodies in milk than in serum [24]. In this regard, bulk tank milk (BTM) sample analysis has been used successfully in surveys of the herd prevalence of several bovine diseases, including coxiellosis [22,25,26,27]. It has been stated that ELISA applied to BTM samples had a sensitivity of 91% in terms of detecting herds positive for *C. burnetii* [28]. Currently, BTM is being used as the basis of herd testing in the Swedish and Swiss national eradication programs for bovine viral diarrhea [29,30,31,32].

Considering the information outlined above, the aim of the present study was to determine the prevalence of *C. burnetii* among dairy herds in the NW of Spain using ELISA bulk milk testing. In addition, the second objective was to determine the on-farm risk factors associated with the exposure to *C. burnetii*, along with its effect on reproductive performance.

## 2. Materials and Methods

### 2.1. Animals and Milk Sampling

BTM samples were collected from 262 commercial dairy herds, including 12,052 Holstein Friesian cows from NW Spain. Sample size was calculated to obtain a confidence level of 95% and a margin of error of 10%. The population size was ~7000, according to the number of dairy farms in the NW Spain. Therefore, a total of 95 farms were needed. From this number, the maximum number of herds were included, decreasing the margin of error to 6%. Farms were conveniently selected from within the client lists of seven veterinarians who collaborate with the Universidade de Santiago de Compostela (USC). Routine reproductive examinations, with data collection and curation in a management software, the membership of a livestock health protection association (ADSG), and their willingness to participate in the study were set as inclusion criteria. The herds were located in the provinces of Lugo, A Coruña, and Pontevedra, and housed an average of 46 cows (8–305). The farms were classified according to their stabling management as follows: free stall, free stall + pasture, free stall + exercise area, tie stall, tie stall + pasture. All farms had a conventional milking parlor, and the cows were milked 2–3 times a day, depending on the type of farm management.

Samples were collected from late January to early April. After stirring the contents of the tank to obtain a homogeneous sample, 50 mL of BTM per farm was collected into sterile plastic tubes, placed into a refrigeration (4–8 °C), and sent to the laboratory of the reproduction and obstetrics unit at the Faculty of Veterinary Medicine of USC (Lugo, Spain). There, the samples were frozen (−20 °C) until posterior analysis.

### 2.2. Sample Analysis for the Diagnosis of Coxiellosis

A commercial indirect ELISA LSIVET RUMINANT Milk/Serum Q FEVER kit (CoxLS kit, Laboratoire Service International, Lissieu, France), previously validated for use for bulk milk testing [33], was used to determine the presence of antibodies against *C. burnetii* in the milk samples. The test was carried out according to the manufacturer’s instructions. In short, milk samples were diluted 1:20 in dilution buffer and 100 µL was placed into the 96 wells of the ELISA plates, which were coated with antigen. The samples were incubated at 4 °C during the night, washed four times, and incubated again with 100 µL of anti-ruminant IgG peroxidase conjugate for 1 h at 37 °C. The plates were washed another four times, and the wells were incubated for 10 min at 22 °C in darkness with 100 µL of the substrate tetramethylbenzidine. The colorimetric reaction was stopped by adding 100 µL of 0.5 M H_2_SO_4_. The antigen used with the ELISA CoxLS kit was isolated from domestic ruminants at INRA, Nouzilly (France). A mix of both antigen phases (I and II) was used in this assay to detect total anti-*C. burnetii* antibodies [34]. For each sample, the S/P ratio was calculated as follows:

$$S/Pratio=\frac{OD\;sample-OD\;negative\;control}{OD\;positive\;control-OD\;negative\;control}\times 100$$

where *OD* = optical density.

The results were expressed as titers (titer = S/P per cent). The S/P titer was categorized in four semi-quantitative groups: negative (S/P ≤ 30), weak positive (+; 30 < S/P ≤ 100), positive (++; 100 < S/P ≤ 200), and strong positive (+++; S/P > 200). Additionally, each BTM sample was scored qualitatively as negative or positive for antibodies against *C. burnetii* when the titer was ≤30 and >30, respectively, as recommended by the supplier.

### 2.3. Data Collection

During the farm visits, a survey (Table S1) was conducted to collect information from farmers and herd veterinarians about potential risk factors. Data collected included details about the geographical area (the province where the farm was located), herd size, housing type, average age of the animals, youngstock management (raised at the farm or custom raised), type of breeding (all artificial insemination (AI) or combined with mating), and the purchase of livestock from other herds.

Additionally, reproductive data from each farm were provided by a collaborator veterinarian, who collected all the information using the reproductive software. Information about the calving to first AI interval, conception rate and first-service conception rate (FSCR), days open, heat detection rate, incidence of abortion, and culling rate was gathered during the year prior to the start of sample collection. Moreover, the prevalence of metritis (abnormally enlarged uterus and fetid watery red–brown uterine discharge, associated with signs of systemic illness and fever, within 21 days postpartum) and endometritis (an inflammatory process of the endometrial lining of the uterus, accompanied by a purulent or mucopurulent vaginal discharge, in the absence of systemic signs of illness, 21 days or more postpartum) was determined [35]. Furthermore, the somatic cell count (SCC), provided by the monthly reports of the regional dairy herd improvement association (DHI), was also registered.

### 2.4. Statistical Analysis

Farms were classified according to the BTM ELISA results (negative: ≤30 or positive: >30), and these were considered to be categorical variables. Similarly, the geographical area (A Coruña = C, Lugo = L, and Pontevedra = P), herd size (≤36, 37–60, >60), stabling management (free stall, free stall + pasture, free stall + patio, tie stall, tie stall + pasture), youngstock management (at the farm or custom raised), use of bulls (yes or no), and purchase of livestock from other herds (yes or no) were considered categorical variables. On the other hand, average age, SCC, calving to first AI interval, conception rate, first-service conception rate, days open, culling rate, and the incidence of metritis, endometritis, and abortion were considered continuous variables.

First, a Pearson’s χ^2^ test was carried out to assess the relationship between seropositivity to *C. burnetii* and the risk factors evaluated at each farm, in order to preselect the significant variables. Thereafter, to identify which risk factors best differentiate herds according to their health status, a classification tree analysis was performed with a farm as the observational unit, using *C. burnetii* seropositivity as the dependent variable and geographical area, herd size, stabling management, average age, youngstock management, use of bulls, and purchase of livestock from other herds as independent factors. Due to the low number of herds located in the province of Pontevedra and its proximity to Lugo, both provinces were considered as one geographical area in the classification tree analysis.

Additionally, the impact of seropositivity to *C. burnetii* on conception rate and FSCR was analyzed using a one-way ANOVA test to verify if there were significant differences between the mean values of positive and negative herds. The analysis was performed using the general linear model (GLM) tool, including conception rate and FSCR as dependent variables, and the *C. burnetii* seropositivity as a factor. Homoscedasticity was checked using the Levene test (*p* > 0.05), and normality was tested using kurtosis and asymmetry (values ranging from −0.5 to 0.5).

Moreover, the impact of seropositivity to *C. burnetii* on SCC, calving to first AI interval, days open, culling rate, and the incidence of metritis, endometritis, and abortion was verified using the non-parametric test Mann–Whitney U, including these as dependent variables and *C. burnetii* seropositivity as a factor.

All analyses were conducted in SPSS version 28.0 for Windows (SPSS Inc., Chicago, IL, USA). Differences were considered significant at *p* ≤ 0.05.

## 3. Results

### 3.1. Prevalence of C. burnetii Antibodies in BTM 

**Figure 1:**
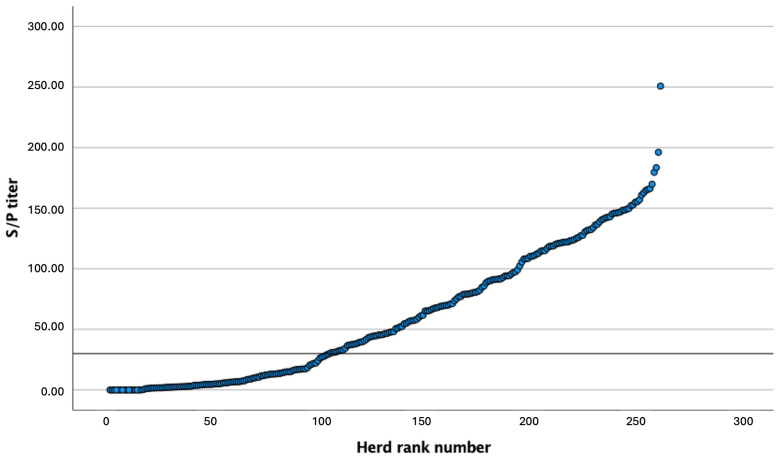
Array of S/P values obtained via the ELISA analysis of bulk tank milk samples to detect antibodies against *Coxiella burnetii* in 262 dairy farms in the northwest of Spain. The grey line indicates the S/P titer 30, considered the threshold between positive (>30) and negative farms (≤30).

**Figure 2:**
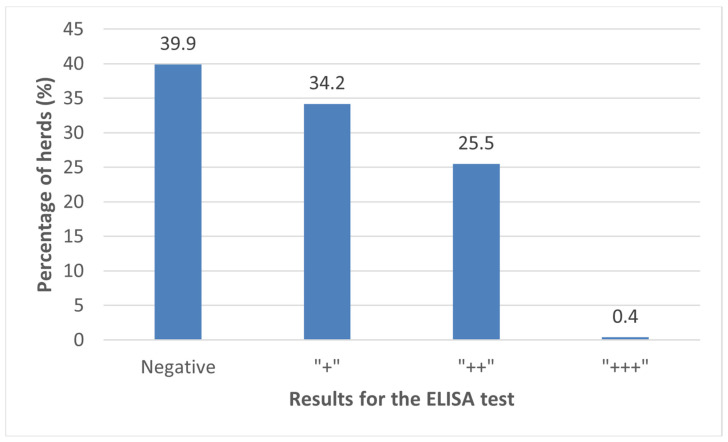
Display of the results of the ELISA analysis of bulk tank milk samples to detect antibodies against *Coxiella burnetii* in 262 dairy farms in the northwest of Spain, according to the following categorization: negative (S/P ≤ 30), + (weak positive; 30 < S/P ≤ 100), ++ (positive; 100 < S/P ≤ 200), and +++ (strong positive; S/P > 200).

The results of the ELISA values for the 262 samples of BTM from dairy herds in the NW of Spain ranged from 0 to 250 S/P (Figure 1). One hundred fifty-eight (60.1%) of the samples had relative antibody concentrations > 30 S/P, and were, therefore, considered as seropositive for *C. burnetii*. A further breakdown of seropositivity is shown in Figure 2.

### 3.2. Risk Factors

Descriptive statistics are displayed in Table 1. Data from one farm are missing in the statistical analysis due to data loss. The Pearson’s χ^2^ test showed that all the risk factors evaluated, except for the average age of the herd, were associated with *C. burnetii* seropositivity (*p* ≤ 0.05). 

**Figure 3:**
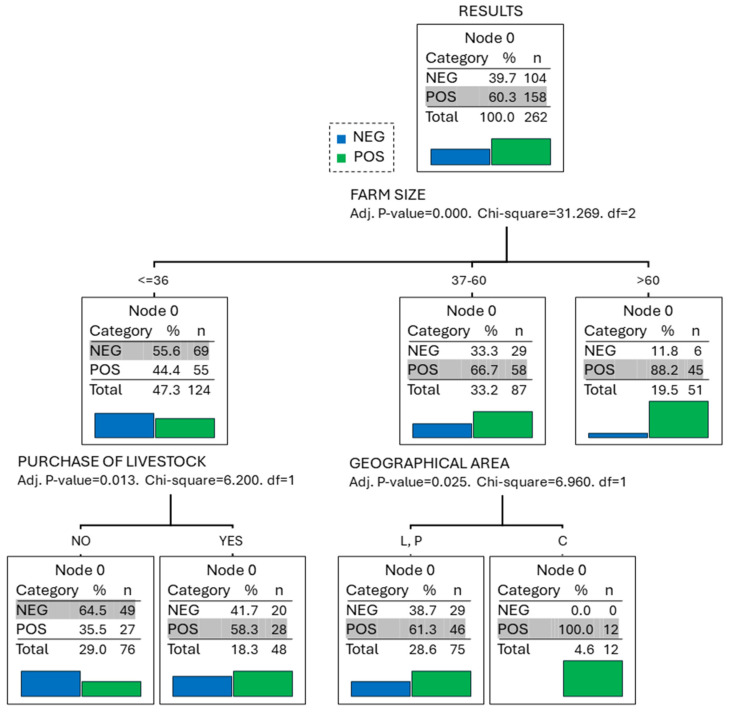
Results of the classification tree analysis including the percentage (%) of dairy farms positive for *Coxiella burnetii* detected in 262 bulk tank milk samples and the risk factor evaluated in dairy farms in the northwest of Spain.

The classification tree analysis identified farm size as the main risk factor for a positive result, with the risk increasing as farm size increases (Figure 3). In this regard, the percentage of farms positive for *C. burnetii* was 44.4, 66.7, and 88.2 for farm size ≤ 36, 37–60, and >60, respectively (*p* < 0.001). The second main risk factor for a positive result, observed only in the small farms (≤36 cows), was the purchase of livestock from other herds. In farms that carried out this practice, 58.3% of BTM samples were positive, while only 35.5% of herds that did not purchase animals were positive (*p* = 0.013). The third risk factor for a positive result, observed only in the intermediate size (37–60 animals), was the geographical area (Table 1), with 61.3% positive farms in the provinces of Lugo and Pontevedra and 100% positive farms in A Coruña (*p* = 0.025).

### 3.3. Relationship between Reproductive Performance and the Presence of Antibodies against Coxiellosis in BTM

Results for the one-way ANOVA test (Table 2) showed that conception rate significantly differed between negative and positive farms to *C. burnetii* (39.8 and 37.1%, respectively, *p* < 0.05). In the same way, significant differences were found for FSCR between negative and positive farms (36.1 and 32.9%, respectively, *p* < 0.05). Regarding the Mann–Whitney U test, a significantly higher incidence of endometritis was observed in positive farms (13.7%) compared to negative farms (11.2%, *p* < 0.05). Additionally, no significant differences were observed for calving to first AI interval, days open, metritis, abortions, and culling rate (*p* > 0.1). As for SCC, no significant differences were observed between negative and positive farms (316.9 and 277.5 × 10^3^ cells/mL, respectively, *p* > 0.05).

## 4. Discussion

Even though coxiellosis has traditionally been considered a disease of minor impact on animal production and public health [1], it has been shown that this infection can have a significant impact on both [36], involving significant financial losses associated with the occurrence of reproductive failure in ruminants [37]. Therefore, in recent years, there has been an increasing interest in knowing more about the prevalence and consequences of this disease [38].

The BTM results obtained in our study suggest that the seroprevalence at herd level in the northwest of Spain is 60.1%, higher than the 46.0% observed by Pablos-Tanarro et al. [39] in 2012 in the same region via the analysis of 404 BTM samples using an ELISA assay. Few studies of coxiellosis seroprevalence have been conducted in Spain. In the Basque Country (*n* = 40), a BTM prevalence of 80 and 68% was observed in 2009–2010 and 2011–2012, respectively, with individual seroprevalences of 10.7 and 11.4%, in BTM samples analyzed by ELISA and PCR [40]. In Asturias (*n* = 163) and southern Spain (*n* = 79), the individual seroprevalence, determined by immunofluorescence antibody assay (IFA) and ELISA in cattle blood samples was 18.4 and 39.0%, respectively [18,41]. In Salamanca, Cádiz, Badajoz, Cáceres, Jaén, and Sevilla provinces, 22.0% of the tested animals were classified as positive and herd seroprevalence was 94.0% [39]. In beef cattle, a study conducted in Madrid (*n* = 1100) reported 6.8% of animals as positive after determining the seroprevalence in blood samples using an ELISA assay, and these were identified in 30.0% of the herds [42]. In the Basque Country, an individual seroprevalence of 6.7%, with 43.0% of herds positive was found after the ELISA analysis of 626 blood samples [43]. The lower individual prevalence observed in beef cattle could be explained by the semi-extensive management conditions under which animals are moved in large areas during part of the year, which reduces contact among animals, as has been seen in other studies [18]. Additionally, *C. burnetii* seroprevalence values in beef cattle were similar for heifers (1–3 years) and adults (>3 years) [18], although it has been reported that the pathogen contact rate tends to increase with age as a consequence of the increasing likelihood of contact with life span [18], as has been observed in dairy cattle [44].

As was stated in the introduction, serological studies conducted at herd and animal levels in other countries reported seroprevalences ranging from 0.0% to 97.2% [10,22,23]. In England, Wales, the south of Italy, Portugal, Nigeria, and the countries from the Eastern Mediterranean Region, seroprevalences of 21.0, 35.0, 20.0, and 20.3% were observed [22,45,46,47,48]; lower than that obtained in our study. Conversely, in the United States, Hungary, and the Czech Republic, higher prevalences were reported, with 90.0, 97.2, and 91.6% of herds being positive, respectively [23,49,50]. In Denmark, Iran, Pakistan, and Belgium, prevalences similar to ours were observed, with 59.0, 56.8, 58.9 and 57.9% of herds being positive [27,51,52,53]. However, comparisons between studies using different methodologies, including different samples (blood or milk), criteria (individual or herd level), and diagnostic tests (ELISA, IFA, or PCR), are difficult to interpret [54], which highlights the importance of conducting new research to update information regarding this disease. Moreover, one limitation of our study is that, because we determined the seroprevalence using an ELISA test and BTM samples, we do not really know which animals are affected, thus the seropositivity of the herd might be due to one positive and actively infected animal, especially in small farms, or to several cows that were infected in the past.

Additionally, in our study, the geographical area, larger farms, sending heifers to a custom raising facility, the purchase of animals outside the farm, performing natural mating, and housing animals in a free stall regime were identified as risk factors, according to the Pearson’s χ^2^ test. Only average age was not associated with farm seropositivity. Moreover, the classification tree analysis selected large herd size, purchasing animals, and the geographical area as the main risk factors related to *C. burnetii* seropositivity. The positive association of herd size with coxiellosis in cattle was also reported by other researchers [10,18,46,55]. In contrast, Taurel et al. [56] and Nokhodian et al. [27] observed higher seropositivity in small farms. It has been stated that this association was stronger in dairy herds [57]. This observation may be explained by the increased probability of transmission and the persistence of the bacteria in the herd once introduced correlating with a growing number of cows in the herd and the higher levels of confinement in these large dairy herds [58], especially due to the concentration of calvings, a critical moment for the transmission of *C. burnetii*. Larger herds may have more contact with the outside and this could facilitate the introduction of the bacteria into the herd [10]. 

Another risk factor that was associated with coxiellosis was buying animals from outside the farm. It should be noted that this association was only significant in small farms (<36 cows). It has been reported that the purchase of animals increases the risk of introducing *C. burnetii* in the herd [36]. The higher risk in small farms may be related to a less rigorous animal management, including poorer selection of animals, the absence of quarantine and the lack of health status monitoring of purchased cows before their introduction into the herd.

Another risk factor included in the multivariable model was the geographical area, being significant in medium size farms (37–60 cows). In this regard, 100.0% of farms located in A Coruña province tested positive for coxiellosis. However, it should be borne in mind that only 12 medium size farms from this province were included in the study, compared to the 75 farms from Lugo and Pontevedra, which may impair the statistical power of the model. Geographical differences could also be related to the density of cattle in the different geographical areas and to the effect of climate conditions [10,58]. Some studies confirmed that the amount of precipitation is inversely proportional to the incidence of coxiellosis, with rain acting as a protective factor by reducing the dust and the aerosolization of *C. burnetii* [59]. A marked variation in seroprevalence between different geographical areas of Northern Ireland have also been observed by McCaughey et al. [57]. 

Although the remaining risk factors were not included in the multivariable model, they are mentioned in the literature in relation to seropositivity in dairy farms [10,42,59,60,61,62,63,64]. In our study, the preliminary analysis suggested that there was greater probability of positive results when youngstock was sent to a heifer custom raising facility. In the same way, the use of bulls increased the risk of coxiellosis. This can be explained by the higher probability of bulls being purchased from other herds [65], which may introduce infection to the farm. Additionally, the interaction of multiple cows might make the bull a vector for infection, as has been reported for sheep [64]. In terms of custom raising, the explanation would be similar, as they can acquire the infection in at external center. This situation might be comparable to the purchase of heifers, another risk factor that may increase seropositivity in dairy herds [42].

Moreover, the percentage of seropositive farms varies depending on housing type. Regarding free-stall farms, 58.0–72.0% are positive, while the incidence decreased to 47.0–51.0% in tie-stall herds. The disparity in the occurrence of coxiellosis in cattle between different housing regimes was also reported by Czaplicki et al. [60] and Neare et al. [10]. These researchers found that cows in loose housing systems had a higher probability of being positive than cows in tie-stall management. The most probable explanation for this is the increased contact between uninfected and infected animals, which facilitates the dissemination of *C. burnetii*.

Furthermore, no effect of average age was found in our study. Similarly, no age-related effect was detected in other studies performed on beef or dairy cattle [43,66]. Although it has been reported that older animals are more at risk of being seropositive [10,42,61,62], it should be noted that in our study we compared the average age of the herd and BTM positive results, while other studies compare the age and seropositivity of individual animals. The association between the seropositivity of the herd and the average age of the cattle could be explained because the older the cow, the more likely she is to have been in contact with infected animals [10]. In previous studies, it was reported that the highest individual seroprevalence was in cows more than 5 and 4 years of age, respectively [61,67].

Concerning reproductive performance, our results showed an association between the depletion of fertility and the presence of antibodies against *C. burnetii*, in contrast to what other studies have discovered [65,68]. Similar data were reported by López-Gatius et al. [69]. The depletion of fertility could be explained by several factors. It has been stated that positive cows have almost twice the likelihood of having retained placenta [49,68]. However, one study carried out by Garcia-Ispierto et al. [70], did not establish a relationship between retention of placenta and coxiellosis. It has been hypothesized that the association between the presence of *C. burnetii* and the occurrence of retained fetal membranes may be due to the placentitis caused by the bacteria, resulting in mild cotyledonary changes in the affected animals [71,72]. Another factor involved in the reduction in fertility is the increased incidence of uterine disease in positive herds [14]. In our study, an increased incidence of endometritis has been observed, which agrees with previous research [11,15,73]. It is known that uterine pathology plays a primary role in the decline in reproductive efficiency, being related to delays in postpartum return of ovarian activity, decreasing pregnancy rates, increasing number of services per conception, and higher culling rates [74,75,76]. Additionally, it is important to consider that reproductive conditions such as retained placenta act as risk factors for the occurrence of other diseases such as metritis, endometritis, mastitis, and lameness [77,78,79], which, in turn, also impair reproductive performance. Moreover, fertility in positive cows is also reduced by the higher risk of pregnancy loss during the first trimester in comparison with non-infected cows [69]. No difference in the number of abortions between positive or negative herds has been identified in our study. This fact contrasts with a study carried out by Bildfell et al. [72], but agrees with several other previous studies [79,80]. Finally, we did not observe a significant influence on calving to first AI interval and days open, which agreed with previous research [68]. It is important to note that reproductive efficiency can be affected by multiple factors, and some of them might be difficult to control. Therefore, the negative effects observed in this study must be considered and further research should be conducted to elucidate the actual role of coxiellosis as a potential factor of reproductive impairment.

Likewise, apart from the multiple events that can worsen reproductive performance, such as the above-mentioned diseases and inappropriate animal management practices, it is important to consider the genetic diversity of *C. burnetii*. Piñeiro et al. [40] observed up to 15 different genotypes from 60 BTM and 7 dust samples, including 7 genotypes reported for the first time. Therefore, the characterization of the genotype present at a farm could be interesting to study, as could the different genotypes of *C. burnetii* and their effects on reproductive performance.

Finally, our data did not report an association between SCC and coxiellosis, contrary to what other studies have described [52,63,80]. It is probable that the fact of detecting the prevalence of coxiellosis and determining the SCC at herd level, instead of an individual level, led us to different results compared to the other studies mentioned. All farms included in our study belong to an ADSG and were also monitored by the DHI association. This implies a rigorous control of the herds regarding heath status, welfare, and maintenance of facilities. As for mastitis surveillance, the DHI association performs monthly collections of milk samples from each lactating cow of the herd, which allows the detection of subclinical mastitis, the determination of its incidence at a farm, and the implementation of appropriate preventive measures. Therefore, the individual cases of subclinical mastitis could be minimized and would not substantially influence the mean SCC of the herd.

## 5. Conclusions

It can be concluded that there was a high prevalence of dairy herds positive for *C. burnetii* in the NW of Spain, indicating that some of these herds may be experiencing infection. The main risk factors identified were farm size, the purchase of livestock by small-sized farms, and geographical area for intermediate size farms. Moreover, positive farms had a decrease in fertility and a higher incidence of uterine pathology compared to negative farms. Further research is needed, including the characterization of the genotype and the identification of active infection in positive herds using direct diagnostic techniques, in order to evaluate the risk factors and true relevance of *C. burnetii* infection in the NW of Spain.

## Figures and Tables

**Table 1:** Results for the Pearson’s χ^2^ test including the number of dairy farms positive for *C. burnetii* detected in 262 bulk tank milk samples and the potential risk factors evaluated in the study.

Variable	Groups	Seropositivity (%) (Seropositive/Total)
Geographical area	C ^a^	80.0 (28/35)
	L ^b^	56.8 (126/222)
	P ^a,b^	80.0 (4/5)
Herd size (number of cows)	≤36 ^a^	44.4 (55/124)
	37–60 ^b^	66.7 (58/87)
	>60 ^c^	82.2 (45/51)
Average herd age (years)	≤5 ^a^	64.1 (109/170) ^†^
	>5 ^a^	52.7 (48/91)
Purchase of livestock	No ^a^	53.7 (79/147)
	Yes ^b^	68.7 (79/115)
Housing type	Free stalls ^a^	72.3 (73/101)
	Free stalls + pasture ^a,b^	58.6 (17/29)
	Free stalls + exercise area ^a,b^	63.6 (14/22)
	Tie stalls/Stanchion barns ^b^	51.0 (26/51)
	Tie stalls/Stanchion barns + pasture ^b^	47.5 (28/59)
Heifer raising	At the farm ^a^	59.4 (152/256)
	Custom raised ^b^	100 (6/6)
Use of bulls	No ^a^	56.0 (117/209)
	Yes ^b^	77.4 (41/53)

^a^,^b^,^c^ Different letters indicate statistically significant differences among groups within the same variable. ^†^ Data about average age was missing for one farm.

**Table 2:** Results for the one-way ANOVA and Mann–Whitney U test including the number of dairy farms positive for *Coxiella burnetii* detected in 262 bulk tank milk samples and the reproductive parameters and pathologies evaluated in dairy farms in the northwest of Spain.

Variable	Positive Farms (±SD)	Negative Farms (±SD)
Abortions (%)	9.79 (±9.36)	8.91 (±8.12)
Metritis (%)	11.52 (±9.37)	10.22 (±8.26)
Endometritis (%) *	13.73 (±9.25)	11.23 (±9.34)
Culling rate (%)	26.55 (±11.95)	27.87 (±16.7)
FSCR (%) *	32.90 (±11.80)	36.13 (±13.16)
CR (%) *	37.07 (±10.28)	39.78 (±11.72)
Calving to first AI interval (days)	78.50 (±14.06)	84.99 (±16.03)
Days open	153.11 (±32.36)	154.03 (±36.36)
SCC (×103 cells/mL)	277.49 (±142.03)	316.92 (±137.89)

* *p* ≤ 0.05; FSCR: first-service conception rate; CR: conception rate; SCC: somatic cell count.

## Data Availability

Data generated and/or analyzed during the current study are available from the corresponding author on reasonable request.

## Supplementary Materials

The following supporting information can be downloaded at: https://www.mdpi.com/article/10.3390/ani14030367/s1, Table S1: Survey designed for data collection in dairy farms tested for coxiellosis in the northwest of Spain.
