# Supplementary material for: Prevalence, Risk Factors, and Relationship between Reproductive Performance and the Presence of Antibodies against Coxiellosis in Dairy Farm Milk Tanks in the Northwest of Spain
Source: Animals (Basel). 2024 Jan 23;14(3):367. doi: 10.3390/ani14030367 (PMC10854522; doi:10.3390/ani14030367)
Supplement: Supplementary file 1 [file animals-14-00367-s001.zip › animals-2783704-supplementary.pdf]

Supplementary materials

Table S1: survey designed for data collection in dairy farms tested for coxiellosis in the Northwest of Spain.

**Coxiellosis: survey for intensive regime dairy farms from Galicia**

**Date**	**Interviewer**
**Farm data**	
Name of the farm:	
Register number:	
Province:	
Village:	

**Question**	**Answer**
**Number of milking cows:**	
**Housing type:**	○ Tie stall<br>○ Free stall<br>○ Other: _________
**Milking management**	○ Milking parlor<br>○ Automatic milking system
**Youngstock management**	○ Raised at the farm<br>○ Buying animals<br>○ Custom raised
**If buying animals, are these purchased out of Galicia?**	○ Yes<br>○ No
**When was the last purchase and how many animals?**	
**Is there a calving pen?**	○ Yes<br>○ No
**Calving pen capacity**	○ Individual

	○ 1-3 animals<br>○ >3 animals
**Is there a quarantine pen?**	○ Yes<br>○ No
**Location of the quarantine pen**	○ Other stall<br>○ Same stall
**Is there a pen for sick animals?**	○ Yes<br>○ No
**Presence of other species in the farm:**	○ Small ruminants (goats, sheep)<br>○ Dogs<br>○ Cats<br>○ Domestic birds<br>○ Swine
**In case of presence, are they in the same pen?**	○ Yes<br>○ No
**Is there any contact with animals from other farms?**	○ Yes<br>○ No
**Is there any contact with wild ruminants?**	○ Yes<br>○ No
**Is the farm member of a livestock heath protection association (ADSG)?**	○ Yes<br>○ No
**Name of the ADSG:**	
**ADSG level → Bovine viral diarrhoea (BVD)**	○ Level 0<br>○ Level 1<br>○ Level 2<br>○ Level 3
**ADSG level → Infectious bovine rhinotracheitis (IBR)**	○ Level 0<br>○ Level 1<br>○ Level 2<br>○ Level 3<br>○ Level 4<br>○ Level 4+

**ADSG level → paratuberculosis**	○ Level 0<br>○ Level 1<br>○ Level 2<br>○ Level 3<br>○ Level 4<br>○ Level 5<br>○ Level 6
**ADSG level → Neosporosis, outbreaks in the last year**	○ Yes<br>○ No<br>○ Not available
**Use of bulls for breeding:**	○ Yes<br>○ No
**Who performs the artificial insemination?**	○ Owner<br>○ Veterinary<br>○ Other: _________
**Were there any reproductive problems in the last year?**	○ Yes<br>○ No<br>○ Not available
**Which type:**	○ Abortion<br>○ Metritis<br>○ Placental retention<br>○ Other: _________
